# Global Advances in Value-Based Payment and Their Implications for Global Health Management Education, Development, and Practice

**DOI:** 10.3389/fpubh.2018.00379

**Published:** 2019-01-18

**Authors:** Michael A. Counte, Steven W. Howard, Larry Chang, William Aaronson

**Affiliations:** ^1^Department of Health Management and Policy, Saint Louis University, St. Louis, MO, United States; ^2^Taipei Beitou Hospital, New Taipei, Taiwan; ^3^Department of Health Services Administration and Policy, Temple University, Philadelphia, PA, United States

**Keywords:** hospital performance, hospital quality, value-based payment, health care management education, healthcare costs, pay for performance, health policy

## Abstract

Global advances in health policy reform, health system improvement and health management education and practice need to be closely aligned to successfully change national health policies and improve the performance of health care delivery organizations. This paper describes the globally acknowledged need for incentive-based organizational performance and relevant implications for health care management education (HCME) and practice. It also outlines the major rationale underlying Value-Based Payment (VBP) or Pay for Performance (P4P) health policy initiatives and their basic elements. Clearly, the major global health policy shift that is underway will likely ultimately have major impacts on the strategic and operational management and performance of health care delivery organizations. Thus, practical specific suggestions are made regarding changes that need to be introduced and strengthened in contemporary health care management education and development programs to help organizational managers in the future.

## Background and Rationale

There is a growing recognition around the global that managers of healthcare organizations must receive management training to be effective systems leaders. This paper focuses on a major trend in health system reform that has direct implications for the types of skills and competencies that health system managers must acquire to assure effective healthcare organizational and health system performance. We address the need for increased curriculum focus on the underlying competencies that managers need to acquire in order to respond to the incentives and expectations that are built into health financing systems that will increasingly depend on value-based approaches to budgeting and payment.

### Goals of This Paper

We first discuss the rapid, global change of health care delivery reimbursement models and methods and the strain that is being experienced in many health systems because of rising health care costs and persistent systemic problems such as inefficiency and variable effectiveness. The major purpose of this paper is to explore the global interaction of reimbursement changes, global health management education, training programs, and health management practice.

Many health systems are experimenting with or implementing payment systems that rely on pay-for-performance (P4P) or value-based payment (VBP). The trend in health system payment in moving from pay-for-quantity to pay for quality. Changes in the incentive structures require radically different organizational goals and strategies in order to maintain fiscal viability. Current managers will need re-education and new managers will need to be well-trained in meeting the new economic environment they are facing.

Two areas that we address in this paper are:

1. What changes in management practice will be needed of future health care managers and leaders to help their organizations effectively respond to the full-scale introduction of P4P/VBP? Will there be changes needed in traditional management development programs, so they are not “globally blind” to useful practices in other countries (1)?
2. How do HCME programs across the world need to change to fully ensure that their graduates are well-prepared for successful employment in healthcare organizations operating in the new financial environments?

While we focus specifically on healthcare management education, it is important to recognize that the changes in the system of financing discussed here have far-reaching implications for health professional and public health education. Health professionals (physicians, nurses, therapists, health records managers, etc.) must be prepared to work in an environment where they are held increasingly accountable for the quality they deliver and impact they have on the health of populations.

### Drivers of Global Changes in Health Care

Although there is considerable variation in health system organization, ownership, and payments across the world (2), it is clear that all health systems are facing major macro-level drivers of change. These drivers include the rapid diffusion of health care information systems, aging populations, increased demand for medical treatments, and widespread recognition that health care systems (and their constituent provider organizations—ambulatory care facilities and hospitals) must significantly and continually improve their performance. However, healthcare organizations are increasingly able to collect, organize, and analyze large amounts of data generated by electronic health records (EHRs). Thus, they are developing the capacity and capability to meet the demands of the new payment environments.

### Payment for Personal Health Care Services

As Jacobsen (3) has noted, the World Health Organization reports that the diverse types of health care financing systems can be contrasted across four different domains. They are: (1) the sources of funds, (2) payment of services, (3) risk/cost burden, and (4) level of coverage. In the last 20 years, international health care system reform efforts have accelerated for four interrelated reasons. First, it has been widely recognized that health care systems need to increase the value of their efforts and outcomes (return on investment and sustainability). Second, there is widespread agreement that there is too much of an emphasis upon rewarding the volume vs. the quality and safety of health care delivery. Third, performance assessment, and ultimately improvement, must take full advantage of new sources of data and analytic methods. And fourth, patient concerns and reports of their personal experiences and satisfaction need to be fully acknowledged and incorporated into the payment of health care services.

### Major Challenges to Funding and Controlling Health Care Costs

Given the continued escalation of health care expenditures across national health care systems, as initially reported by Savage et al. (1), there have been many different types of remedial health policy initiatives. They have included balancing levels of private and public payment, mixing non-profit and for-profit providers and creating mixtures of market forces and regulations. Also noteworthy are experiments with new payment structures, managed care designs, changes in budgeting, capitation, and many other types of regulatory interventions. However, as the authors conclude: “While successful at improving the availability of care, attempts to remedy rising costs have failed. They suggest that policy reforms can only address certain aspects of the iron triangle of access, cost and quality…Comparative studies of international health care management …need to delineate innovative ways to improve the quality and the efficiency of health care delivery.”

Because of the problems cited above, health care reform efforts continue to be implemented across the globe. They are typically sensitive to local constraints. Nonetheless, there is a common strong focus across national health care systems on:

- Broadening individual insurance coverage and reducing barriers to access;
- Improving levels of unwanted variation of quality and safety;
- Shifting the focus from sickness to wellness care; and
- Lowering the costs of health care services.

## Introduction of Pay-for-Performance/Value-Based Payment for Health Services

The basic idea of linking the level of financial payment for a health care service to the quality of the provider's service has been of long-standing interest to health care policy makers. The movement began in highly developed countries, but with strong support from international organizations such as the World Bank, it is now also being extended to lower-income countries (4).

### Fee-for-Service vs. Salaried Physicians

During health care reform policy discussions and initiatives, a variety of reasons are offered for why certain health care systems tend to have much higher costs. Underlying reasons are often cited to be administrative costs and practice patterns of fee-for-service (FFS) physicians that lead to “over-treatment” of patients. However, even integrated, capitated health plans (the obverse of FFS) have also been criticized because of their predilection to “cherry pick” healthier populations and avoid sicker ones (5).

**Figure 1:**
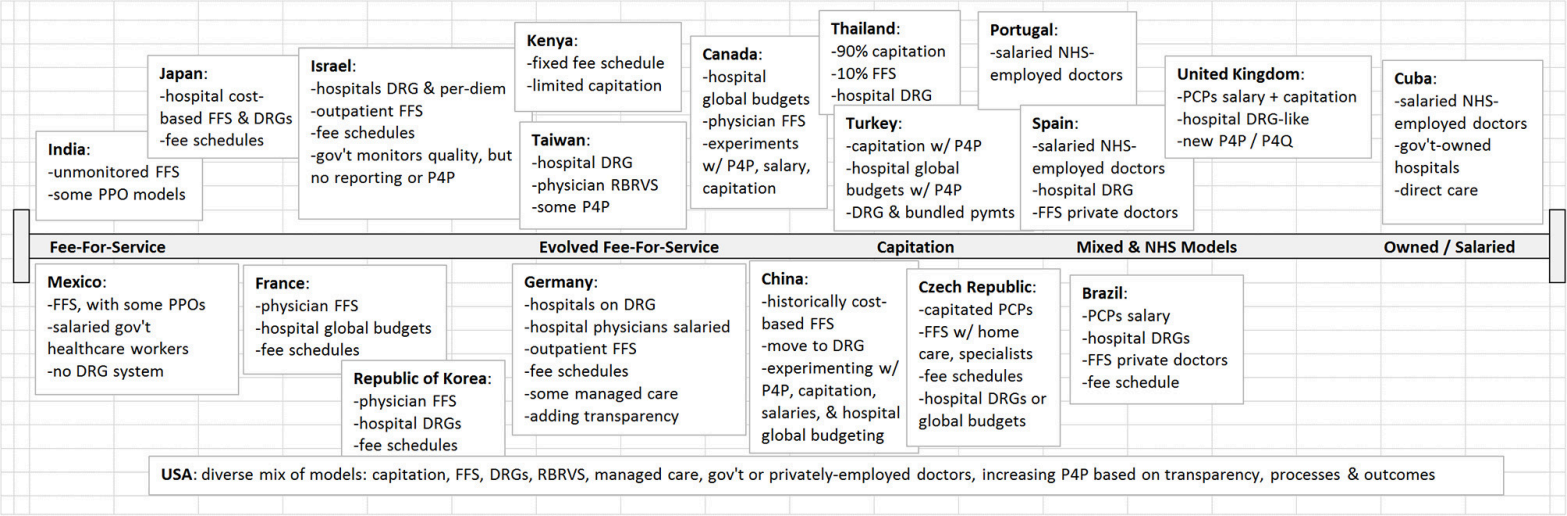
Healthcare Payment Models in 20 Countries. Adapted from Fried and Gaydos (2). FFS, Fee for Service; DRG, Diagnosis-Related Groups; PPO, Preferred Provider Organization; RBRVS, Resource-Based Relative Value Scale; P4P, Pay for Performance; P4Q, Pay for Quality; NHS, National Health Service; PCP, Primary Care Provider.

Figure 1, based on the findings of Fried and Gaydos (2), depicts the diverse approaches to provider payment observed in 20 different countries spanning the globe. While nearly all have at least some use of FFS, we see evidence of low, middle, and high-income countries experimenting with multiple alternative means of payment, including forms of P4P.

### Introduction of Pay-for-Performance (P4P) in Health Care

**Figure 2:**
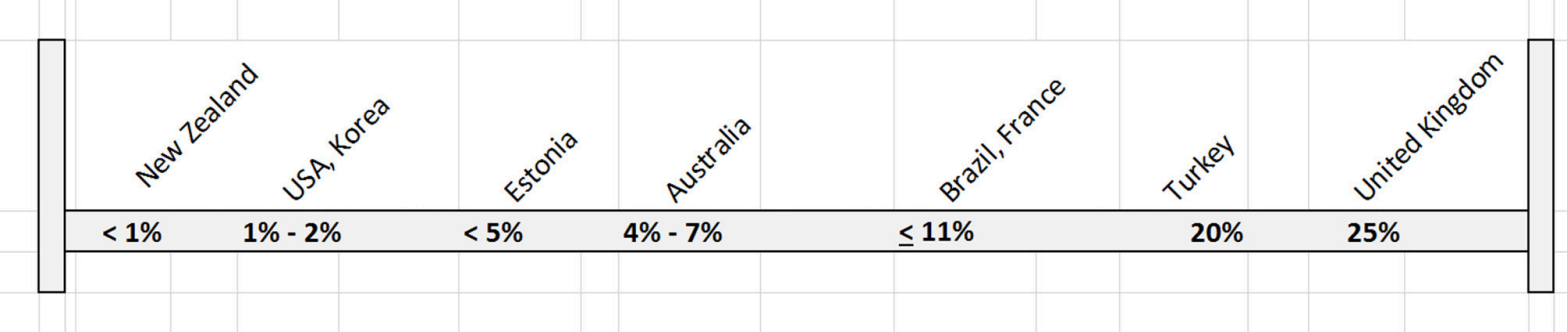
P4P as Percentage of Base Payments. Adapted from Cashin et al. (8). In all cases presented, P4P was in the form of bonus payments or withhold models. None in the form of penalties. German P4P system pays a flat rate. Maryland USA system pays a variable amount not determined. Both were omitted from this Figure.

Beginning in the mid-1990s, a series of influential reports were produced by the Institute of Medicine in the US that ultimately led to increased attention to the issues of the quality and safety of patient care in America (6, 7). What the IOM reports basically documented were referred to as significant “quality gaps” between physician practice patterns vs. best-practices supported by evidence. A recent major OECD report addressed the widespread diffusion of P4P across ambulatory care providers and hospitals in Europe, Brazil, Korea, Australia, New Zealand, and the US between the late 1990s and 2010 (8). This very ambitious and comprehensive report highlighted that the general idea of “paying for results” has attracted substantial interest across the world since most health systems are facing ever-increasing health costs which continue to further strain budgets and fuel interest in “trying to obtain more for less” (improved health care processes and personal health service outcomes for lower costs). While global interest in P4P is rising, the extent to which experimenting countries use it to influence overall provider compensation is highly variable. This shown below in Figure 2.

Despite the attractiveness of the concept of P4P, meta-analysis of the reported effects of P4P indicate that the effects of financial incentives (especially regarding health outcomes) are very difficult to assess and interpret (9).

### New Perspective: Creating Value-Based Competition on Results

Porter and Teisberg (10) introduced a new approach to improving health care by focusing on the structure of health care delivery itself. Their general thesis is that in normal markets, competition leads to ever-improving quality and lower costs. Alas, they argue that such open competition that will create increasing value for consumers (higher quality/lower costs), is absent from current health care delivery which “erodes quality, fosters inefficiency, creates excess capacity, and drives up administrative costs (11).” Thus, they contend that current ideas such as a focus on provider/organizational practices ala P4P will inherently have limited effects. Instead, a new positive competition needs to be supported that has distinct emphases:

- Value for patients vs. solely cost reduction
- Results-based competition
- Focus on medical conditions over a full cycle of care
- Greater value of provider, experience, expertise and uniqueness of the condition
- Results and price information to support value-based completion
- Incentivize innovations to increase value to the consumer

### Centers for Medicare and Medicaid Services Value-Based Purchasing Program

As stated earlier, many different types of P4P/VBP have been developed across the world. One approach that attempts to incorporate both elements of traditional P4P and the concepts of end-results and consumer value is the Hospital Value-Based Purchasing (VBP) program developed and now implemented in hospitals by the Centers for Medicare and Medicaid Services (CMS) in the US (12–14). The CMS plans to introduce a similar value-based program in ambulatory care organizations (14). Like many P4P programs, it initially sets aside 1%, then eventually 2%, of the total CMS payment pool that can subsequently be awarded to organizations that provide higher levels of service quality and patient safety (~$1.5 billion USD in 2018).

There are four equally weighted VBP Hospital Performance Domains (25% each) and a total of *N* = 24 indicators in the areas of Patient Safety, Clinical Care (select outcome assessment and best-practice compliance indicators), Efficiency and Cost Reduction, and Patient and Caregiver-Centered Experience of Care. Thus, the VB reimbursement system: is a marked departure from FFS incentives; places much more emphasis on results/outcomes; is truly multidimensional since it incorporates both patient and clinical perspectives and is an important part of a major effort to adopt this reimbursement approach in the reimbursement of many if not most types of health services. The goals of these expansive programs are to provide better care for individuals, better health for populations and lower costs.

## Health Care Management Education, Development, And Practice

### Growth and Development of Health Care Management Education and Practice: The Case of Taiwan

During the last 50 years, Taiwan (a small nation of 23 million people in East Asia (closely aligned with the United States, Japan, Australia and Europe) has undergone widespread, dramatic economic development. This broad change has affected all sectors of Taiwanese health care. For example, high economic growth has fostered the development of a very strong educational system, a comprehensive national insurance system called National Health Insurance (NHI) that assures ready access to health care services and finally, high quality health care provider organizations and associations that strengthen the continually improved delivery of health care to its population.

Health care management education programs are of two types. First, there are currently *N* = 11 Master of Health Administration (MHA) programs in Taiwan. The first was offered in 1984 and modeled on the MHA program at the University of Michigan. There are also a smaller number of Health Policy programs offered in Taiwan. There are *N* = 9 undergraduate Health Management programs that have been offered in Taiwan since 1993. Taiwanese health management education programs are often modeled on foreign programs, but modified to meet local needs and cultural expectations. Also, entry into these programs is highly limited since the central Taiwanese government places a strong emphasis on maintaining the quality of academic programs and it limits supply of graduates to the level of current demand from Taiwanese health care organizations (restricts over-supply of graduates).

Pay-for-Performance/Value-Based Payment models are currently being evaluated by the Department of Health in Taiwan and several pilot projects have been initiated. Thus, even though VBP may be an attractive alternative approach to FFS in the NHI, it is being introduced very slowly in Taiwan, and at this point, there has been little inclusion of P4P/VBP in Taiwanese HCME programs. Also, several foreign universities including Saint Louis University, Johns Hopkins University and Tulane University have been active in collaborative HCME programs and the development of extensive management development programs for Taiwanese health care managers and executives.

Taiwan faces important challenges to its health care system in the years ahead. Issues include:

1. The need to adapt to increased needs of a rapidly aging population. This is compounded by a very low birth rate.
2. The financial burden of certain subgroups and the prevalent fee-for-service payment system will necessitate increased regulation to further limit services and payment.
3. Health care labor shortages that create personnel concerns and increased medical disputes.
4. Malpractice claims are rapidly escalating.

For Taiwan to continue to be a major leader in the field of global health care, in coming years it will need to further incentivize health care organizations to become both more efficient and effective, and to fully utilize its population of very highly qualified health care professionals. This will ensure that Taiwan's health care system can continue to improve its already admirable level of performance.

### Impacts of VBP/P4P on Global Health Care Management Education and Development

Like Taiwan, most other middle- and high-income countries are contending with aging populations, low birth rates, and escalating health care costs. Given these demographic and economic trends, health systems that were not previously incentivized to emphasize quality, safety, or value are now being forced to do so. Policymakers are increasingly demanding higher health care quality. Ministries of Health, like that in the Czech Republic, are establishing specialized structures to promote improved safety and quality of care (in Colombia, the Supreme Court has even ordered actions to improve health system quality). However, as observed in Taiwan, the Health Care Management Education (HCME) programs are largely trailing in their responses to health policies that seek to improve value. The Atlas Health Foundation has commissioned multiple assessments of International HCME (15, 16). Together, the Atlas Foundation reports have focused on 22 countries, including the most recent analyses of Colombia, Czech Republic, Germany, Ireland, the Netherlands, and South Korea (16).

While the 2013 report shows an increasing HCME program focus on the teaching of quality initiatives, there was no indication that programs are preparing students to manage the increasing connection between such quality improvement programs and new P4P/VBP payment models. South Korea was ranked as the most advanced environment for HCME, largely because of its strong emphasis on quality improvement. With strong curricular coverage of quality improvement and reimbursement methods, Germany was also highly rated, though the degree of intersection between payment and quality in the classroom is unclear. Irish hospitals have mandatory quality assurance/quality improvement programs and are required to report quality metrics. Further promoting health, prevention, and cost control, Ireland uses a system of general practitioners as gatekeepers. Again, it is unclear that any incentives or penalties are directly tied to quality metrics. The Netherlands may be the most forward-thinking on VBP in Europe. As in Ireland, the Dutch hospitals have mandatory quality reporting. Health insurance is mandatory in the Netherlands and private insurance companies provide the coverage, competing on quality and cost. It is likely this combination that has led to more bundled payments, an early step toward VBP. While South Korea shares other countries' concerns about safety and quality, little has been done to formalize policies at the governmental level. The Korean payment system remains a direct FFS system, and even an effort to institute a relatively modest DRG system failed. Cost is addressed through price-setting by the National Health Insurance Program, but is not tied to quality. Like the U.S., South Korea has common FFS-driven inefficiencies, such as duplication of services and unnecessary utilization.

From these global examples, we see an increasing emphasis on quality and safety, but no consistent trend toward teaching students in HCME programs how to manage quality in an environment that is increasingly linking level of reimbursement to results. Judging from growing interest in the Global Healthcare Management Forum of the Association of University Programs in Health Administration (AUPHA) and the growing number of Global Healthcare Management courses, HCME programs are beginning to recognize this need. Another major step is a forthcoming textbook on Global Health Management on the topic (17). This textbook is dedicated to the topic of Global Health Management education and management development. It focuses on three areas: significant organizational challenges facing health care managers, formulation and implementation of health policies, and macro-level trends that health care organizations will need to adapt to in the future. Clearly the text recognizes the need to adapt to the new emphasis on service value so HCME program graduates are better prepared to manage organizations under the new payment models on the horizon. Yet, even additional changes in the content and delivery of HCME may be in order.

### Changes Needed in Health Management Education

How then should HCME programs adapt their instruction to such important changes in health care reimbursement? First, Health care Performance Improvement (Quality) courses must place greater emphasis on measurement and metrics. For example, as described earlier, the U.S. Centers for Medicare and Medicaid Services has instituted a new VBP program. To succeed under the new VBP program, hospitals must be able to accurately measure and report a group of more than 12 ever-changing metrics (some with sub-components) across four domains: Safety, Clinical Care, Person and Community Engagement, and Efficiency and Cost Reduction. While this is just one VBP program in one country, the catalysts driving this policy are globally relevant. All countries are concerned about improving quality of care, patient safety, and cost reduction. Second, the consumerism trend is also impacting societies globally, with varying degrees of influence over health systems (18). New HCME graduates must be prepared to fully understand these metrics, how health care delivery organizations are affected and ensure health system goals are being met. Health care organization leaders need to be prepared to negotiate target metrics with payers, national health insurance systems, and ministries of health.

## Competencies and Skills for Effective Management Practice

In order to better plan healthcare management educational strategies, it is important to identify the competencies and skills needed to engage in effective leadership and management of healthcare organizations. The environmental changes described in this paper can be addressed through articulation of relevant competencies. The International Hospital Federation with the support of a consortium of professional organizations and educational institutions identified and defined competencies for healthcare leadership that are universally applicable (19).

The competencies required to meet the environmental contingencies fall within two domains: health/healthcare environment, and business. With the health and healthcare environment competency domain, the following health systems and organization competencies are most critical:

- Balance the interrelationships among access, quality, safety, cost, resource allocation, accountability, care setting, community need, and professional roles
- Assess the performance of the organization as part of the health system/healthcare services

In addition, multiple business competencies are required. Special attention must be given to the financial management competencies, especially as follows:

- Effectively use key accounting principles and financial management tools, such as financial plans and measures of performance (e.g., performance indicators)
- Use principles of project, operating, and capital budgeting
- Plan, organize, execute, and monitor the resources of the organization to ensure optimal health outcomes and effective quality and cost controls

### Curricular Issues

The introduction of VBP completely realigns financial incentives. As a consequence, healthcare management educational programs must carefully rethink the traditional approaches to curriculum that focus largely on independence of financial management competencies. There are several specific curricular concerns that need to be addressed in order to assure effective competency development. In addition to updating how we teach Quality, Financial Management curricula must also be modified to be better integrated with Quality Performance. Today, most HCME programs have at least one course in Financial Management. While the basics of Financial Management are as imperative as ever, advanced courses need to be closely aligned with Quality, Operations, Data Analytics, and Customer Experience courses.

Curricular innovation must focus on integrative approaches to program design. By using large, cross-cutting cases and/or live client projects, students can learn the increasing interrelationships between Financial Management and these other functions of the modern health care organization. Negotiation and Leadership courses should use mock negotiation exercises, followed by modeling the financial repercussions of the arrangements negotiated. Then in Analytics class, students learn how to extract data from a database and report on the metrics previously negotiated. In turn, the students can come back to the negotiation table to discuss the outcomes of their previous agreements and propose how they should be modified.

By modifying HCME program curricula to better include the new metric-intensive realities of the changing health care environment, and by better connecting previously “siloed” disciplines, graduates will be better prepared for not only the early stages of their careers, but also for the long-term requirements of health care leadership. Not only will such an approach make HCME programs more relevant to the evolving health care environment, accreditation may well be requiring it. Accrediting bodies, such as the Commission on the Accreditation of Healthcare Management Education require competency-based education. The competencies and approaches to teaching need to be informed by alumni and other external stakeholders from the health care industry. We should expect to see accreditors requiring HCME programs to demonstrate how their models prepare students to succeed in the increasingly interdisciplinary health care work environment where providers' revenues are closely tied to processes and outcomes that produce value for patients and for society overall. These changes in HCME curricula and competencies should also guide future management development programs intended to keep practicing managers and clinicians fully aware of how policy-level incentives will affect the performance of their organizations.

## Discussion and Conclusion

In summary, given the rapid emergence of health policies that promote VBP/P4P, we contend that in the future, health care management education and management development programs need to introduce changes in contemporary health management education and practice. Such programmatic improvements include:

1. Fully explaining the organizational implications of emergent changes in health policy and reimbursement—especially the emerging multi-dimensional view of quality (e.g., clinical, efficiency, patient experience, outcomes, etc.).
2. Placing a much greater emphasis on teaching about quality/process performance management and metrics (both conceptual issues and assessment methods).
3. Acting to vertically and horizontally integrate program curricula (e.g., financial management and operational performance improvement.) Perhaps, this should include cases that students work on across their academic program. This will help students to acquire a systematic perspective that alleviates “siloing.”
4. Ensuring that throughout their program, participants have full exposure to many major changes that are occurring in the real world of health care delivery such as advances in Health Information Technology, the age of Big Data and Analytics, and how effective management interventions can help organizations respond to ever-changing health policy priorities (e.g., Management Rounds, Internships).
5. Helping students to develop and use a strategic management perspective that shows how organizations need to continually learn more about (and perhaps even anticipate) significant micro-level (local area) and macro-level external environmental changes. This is the only way that managers and leaders can effectively modify their organization's appropriate service mix and how quality performance can be used as a source of competitive advantage.
6. Working with other health professional leaders to offer programs that build inter-professional awareness and recognition. These types of experiences should be of value to practice-based attempts to re-structure patient care in alignment with changes in the delivery system.

## Conclusion

This paper outlines the major rationale underlying contemporary VBP or P4P health policy initiatives and their basic elements. Clearly, the major global health policy shift that is underway will ultimately have major impacts on the strategic and operational management and performance of health care delivery organizations. Successful implementation of Evidence-Based Management and ever-improving, complex information systems in Health Care Management Education and Management Development programs will likely provide major benefits to program participants and ultimately, their employer organizations. It is imperative that these changes to HCME and Management Development not happen in a vacuum. While quality improvements and success under P4P/VBP initiatives will require better trained health care managers, clinicians are arguably even more important. Leadership teams at HCME programs around the world must coordinate their efforts with their colleagues in Medicine, Nursing and Allied Health schools. Together, we can achieve much greater impacts on population health and quality health care than we can working in isolation from each other. There are also numerous global health services research implications of VBP/P4P that will provide further useful insights into the dynamics of health policy reform and health system performance during the next several decades.

## Author Contributions

All authors listed have made a substantial, direct and intellectual contribution to the work, and approved it for publication.

### Conflict of Interest Statement

The authors declare that the research was conducted in the absence of any commercial or financial relationships that could be construed as a potential conflict of interest.
